# Influence of Altered Immune-Inflammatory Axis on the Risk of Osteomyelitis and Its Network Interaction Effect in European Population

**DOI:** 10.1155/mi/5707884

**Published:** 2025-04-23

**Authors:** Yangguang Lu, Siyao Chen, Ruotong Yao, Feng Chen, Yukai Wang, Zihui Yuan, Di Lu, Haiyong Ren, Xiang Wang, Bingyuan Lin, Qiaofeng Guo, Kai Huang

**Affiliations:** ^1^The First School of Medicine, School of Information and Engineering, Wenzhou Medical University, Wenzhou, China; ^2^The Second School of Medicine, Wenzhou Medical University, Wenzhou, China; ^3^Department of Orthopedics, Tongde Hospital of Zhejiang Province, Hangzhou, China

**Keywords:** bioinformatics analysis, genetic epidemiology, immune-inflammatory axis, mendelian randomization, osteomyelitis

## Abstract

**Background:** Osteomyelitis (OM) is a severe bone infection with rising incidence rates, particularly among elderly and diabetic patients. The immune–inflammatory axis is implicated in OM pathogenesis, but its complex interplay remains poorly defined. This study aims to explore the causal relationships between immunophenotypes, plasma inflammatory proteins, and OM using Mendelian randomization (MR) and bioinformatics.

**Methods:** Utilizing publicly available genetic data, we undertook a series of quality control measures to identify instrumental variables (IVs) associated with exposure. Subsequently, we conducted MR using inverse variance weighting to explore the causal relationships between immunophenotypes, plasma inflammatory proteins, and OM. Bioinformatics tools were applied to explore the functional enrichment and protein–protein interaction networks of the implicated genes.

**Results:** The MR analysis identified 13 immune cell phenotypes and 2 plasma inflammatory proteins associated with risk of OM. Notably, higher levels of HLA DR on plasmacytoid dendritic cells, memory B cell absolute counts, and CD8dim T cell percentages were associated with increased OM risk. Additionally, elevated levels of CD6 and IL-12 subunit B were correlated with OM risk. Bioinformatics analysis revealed the enrichment of related genes in immune-related pathways and highlighted the complex interaction networks of the implicated proteins.

**Conclusions:** This study provides novel insights into the immune–inflammatory axis in OM and identifies potential biomarkers for risk assessment. The findings warrant further validation in diverse populations and pave the way for developing targeted preventive and therapeutic strategies for OM.

## 1. Introduction

Osteomyelitis (OM) is a severe inflammatory bone disease caused primarily by microbial infections, leading to progressive bone destruction and, in some cases, affecting adjacent soft tissues [1]. OM is closely associated with the body's inflammatory response, with over three quarters of cases attributed to hematogenous infections, predominantly caused by *Staphylococcus* species [2]. Additionally, certain autoinflammatory conditions and genetic predispositions can lead to chronic noninfectious OM, presenting with similar clinical manifestations [3]. Secondary OM may also develop from the spread of infections from nearby sites, orthopedic implants, vascular insufficiency, or neuropathy [4, 5]. The rising incidence of antibiotic-resistant bacterial strains due to misuse of antibiotics has significantly increased the morbidity and mortality associated with OM, particularly among the elderly and diabetic populations, thereby imposing a growing burden on health care systems in aging societies [6, 7]. As such, a deeper understanding of OM pathogenesis is crucial for the development of targeted prevention and treatment strategies.

In recent years, the role of the immune system in OM pathogenesis has gained increasing attention, beyond its well-known links to inflammatory processes. Studies suggest that one of the main reasons for the limited efficacy of current OM treatments is the establishment of an immunosuppressive infection microenvironment [8]. Pathogenic microorganisms employ various strategies to evade the host's immune defense, such as eliminating neutrophils, macrophages, and other antigen-presenting cells, inducing antiinflammatory and pro-fibrotic M2 macrophage responses [9], and promoting the differentiation of myeloid-derived suppressor cells. These mechanisms interfere with T-cell differentiation and activation, induce T-cell apoptosis, and trigger proliferative B-cell apoptosis [10]. Additionally, recent research utilizing a rabbit model of OM has identified immune and inflammation-related biomarkers linked to chronic OM, indicating that immune cells, such as neutrophils and leukocytes, along with interleukin (IL)-6, may serve as diagnostic markers for OM [11]. These changes in immune cell phenotypes, coupled with inflammatory factors, likely form an immune–inflammatory axis that plays a pivotal role in the pathogenesis of OM.

Mendelian randomization (MR) is a powerful epidemiological approach for causal inference, grounded in Mendel's law of independent assortment. It has been widely applied to explore the etiology of various diseases [12]. By leveraging genetic variations that precede disease onset, MR minimizes confounding factors and reduces reverse causation bias in epidemiological studies [13]. In this study, we employed MR and bioinformatics methods to investigate the genetic-level causal relationships between 731 immune cell phenotypes and 70 plasma inflammation-related proteins and OM. Furthermore, we explored the mediating interactions between immune cell phenotypes and plasma inflammatory proteins. Our findings provide novel insights into assessing OM risk and developing preventive and therapeutic strategies.

## 2. Materials and Methods

### 2.1. Study Design

**Figure 1:**
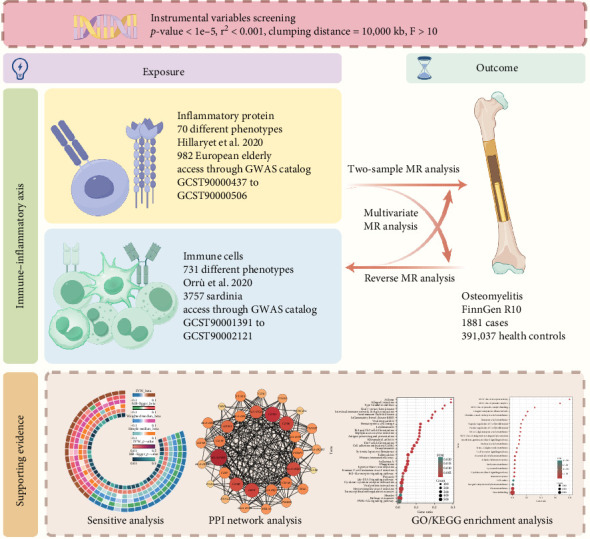
Flowchart of study design and data sources (by figdraw.com).

This study adheres to the Strengthening the Reporting of Observational Studies in Epidemiology Using Mendelian Randomization (STROBE-MR) guidelines (Table S1) [13]. We utilized publicly available genome-wide association study (GWAS) summary data for 731 immune cell traits and 70 plasma inflammation-related proteins. Appropriate single nucleotide polymorphisms (SNPs) were selected as instrumental variables (IVs) for MR analysis to investigate potential bidirectional causal relationships. The application of IVs in MR analysis is based on three key assumptions: (i) the selected IVs must be strongly associated with the exposure of interest, (ii) the IVs must not be confounded by factors influencing the outcome other than the exposure, and (iii) the IVs must affect the outcome solely through the exposure [14]. Additionally, mediation and multivariable MR analyses were conducted, treating immune cell traits and inflammation-related proteins as mediators. Last, bioinformatics methods were employed to explore the protein–protein interaction networks and pathway enrichment of genes related to the findings (Figure 1). All data in this study were derived from previously published research and public databases; therefore, no additional ethical approval was required.

### 2.2. GWAS Data Sources

The immune cell data were sourced from a 2020 GWAS conducted by Orrù et al. [15], which included a cohort of 3757 Sardinian individuals. A total of 731 immune phenotypes were analyzed, comprising 118 absolute cell counts (AC), 389 median fluorescence intensities (MFI) reflecting surface antigen levels, 32 morphological parameters (MP), and 192 relative cell counts (RC). These included traits for B cells, conventional dendritic cells (CDCs), T cells at different maturation stages, monocytes, bone marrow cells, TBNK (T cells, B cells, natural killer cells), and regulatory T (Treg) cells, while MP traits covered CDC and TBNK cells. Detailed study procedures can be found in the original publication. The summary statistics for each immune phenotype's GWAS are publicly available in the GWAS catalog (accession numbers GCST90001391 to GCST90002121).

The plasma inflammation-related protein data were obtained from a 2020 GWAS conducted by Hillary et al. [16], involving a cohort of 876 European individuals. This study employed the BayesR+ Bayesian penalized regression framework, which is particularly sensitive for evaluating complex traits such as plasma inflammation-related proteins [17]. The cohort consisted of older adults, characterized by systemic chronic inflammation, providing a unique profile of plasma inflammation-related proteins [18]. Given that OM predominantly affects the elderly and diabetic populations [19], this data set was selected as the exposure data. Detailed study procedures can be found in the original publication. The summary statistics for each plasma inflammation-related protein's GWAS are publicly available in the GWAS catalog (accession numbers GCST90000437 to GCST90000506).

The genetic data for OM were sourced from the FinnGen consortium (https://r10.finngen.fi/, accessed on Sept 28, 2024), comprising 1881 OM patients and 391,037 non-OM controls. All participants were of European ancestry and provided informed consent. Cases were identified using the International Classification of Diseases, 10th Revision (ICD-10) codes. Details on participant characteristics, genotyping, imputation, and quality control are available on the FinnGen website (https://finngen.gitbook). All genomic locations are based on the human reference genome hg19 (GRCh37). The GWAS data sets used in this study were of European ancestry and free from population overlap, allowing for the appropriate implementation of MR analyses.

### 2.3. Instrumental Variable Selection

To ensure the robustness and reliability of the MR analysis, we applied the following selection criteria for IVs. First, to associate SNPs with the exposure, we set the significance threshold for IVs at *p*< 1 × 10^−5^. Additionally, we removed SNPs in linkage disequilibrium (LD) to avoid bias (*r*^2^ < 0.001, clumping distance = 10,000 kb). In cases of LD, we selected the SNP with the lowest *p*-value for the exposure. Finally, we harmonized the SNPs between the exposure and outcome to ensure consistent effect estimates based on the same effect alleles, and excluded palindromic SNPs with intermediate allele frequencies or SNPs with incompatible alleles.

### 2.4. Statistical Models and Approaches

We used the inverse variance weighted (IVW) method to evaluate the association between exposures and outcomes. The IVW method provides accurate and stable estimates when all IVs meet the three core assumptions of MR analysis [20]. Results were presented as odds ratios (ORs) with 95% confidence intervals (CIs). Heterogeneity was assessed using Cochrane's *Q* test and quantified by the I^2^ statistic. Heterogeneity was considered absent if I^2^ was below 25%, and mild if I^2^ was below 50%. We assessed potential horizontal pleiotropy using the intercept from MR-Egger regression [14]. To ensure robustness, we applied additional MR models to test the sensitivity of the conclusions, including MR-Egger, weighted median, and simple median methods. MR-Egger accounts for horizontal pleiotropy but at the cost of reduced precision [14], while the weighted median method provides consistent estimates when at least 50% of the IVs are valid [21]. For exposures and outcomes that showed causal relationships in the MR analysis, we conducted reverse MR analysis to explore potential bidirectional causality.

Given that we analyzed two data sets (immune cell phenotypes and plasma inflammation proteins), we applied a conservative approach to account for multiple testing. For MR and MVMR analyses, we used a Bonferroni-corrected significance threshold of *α* = 0.025 (0.05/2) to reduce the rate of Type I errors [22]. Additionally, we adjusted the *p*-values using the false discovery rate (FDR) method as a supplementary reference [23]. Negative results that were adjusted using FDR and still met the Bonferroni threshold were considered marginally significant. For the FDR-adjusted data, mediation, and sensitivity analyses, a *p*-value < 0.05 was considered significant. All statistical analyses were conducted using R version 4.3.2 and the “TwoSampleMR” package.

### 2.5. Mediation and Multivariable Analyses

In addition to standard MR analysis, we performed two-step mediation MR analysis to further explore the mediating and interaction effects between immune cell phenotypes and plasma inflammation proteins. For immune cell phenotypes and plasma inflammation proteins showing significant associations (*p* < 0.05) in the initial analysis, we evaluated their roles as mediators influencing OM development through their effects on plasma inflammation proteins or immune cell phenotypes, respectively. Following the methodology applied in previous studies [24], we estimated the direction and proportion of the mediation effect using the following formula based on the *β* values derived from the IVW method:

  $$\begin{array}{c} \theta =\frac{\beta_{1}\times \beta_{2}}{\beta_{0}}, \end{array}$$

where *θ* represents the mediation proportion, *β*_0_ is the direct effect of immune cell phenotypes or inflammation-related proteins on OM, *β*_1_ is the direct effect of immune cell phenotypes or inflammation-related proteins on the mediator, and *β*_2_ is the direct effect of the mediator on OM. The *p*-value for the mediation proportion was calculated using an estimation method based on rates. Complementary mediation effects were defined when the direct and mediated effects aligned in the same direction, while competitive mediation effects were defined when they were in opposite directions [25]. We also conducted multivariable MR (MVMR) analysis to clarify the effects of immune cell phenotypes or plasma inflammation proteins on OM risk after adjusting for mediators, providing additional insights into the role of the immune–inflammation axis [26].

### 2.6. Bioinformatics Analysis

We extracted loci from the GWAS data sets for OM and the immune cell phenotypes and plasma inflammation-related proteins that showed positive associations in the MR analysis. We selected loci that met the genome-wide significance threshold of *α* = 5 × 10^−8^ for subsequent bioinformatics network analyses. Target genes were subjected to Gene Ontology (GO) and KEGG pathway enrichment analysis using the KOBAS tool (http://bioinfo.org/kobas/, accessed on Sept 28, 2024), and key pathways were visualized to explore relevant biological processes, cellular components, molecular functions, and signaling pathways. We further input these target genes into the String platform (https://www.string-db.org, accessed on Sept 28, 2024) for protein–protein interaction (PPI) analysis, and constructed the PPI network using Cytoscape version 3.10.2.

## 3. Results

### 3.1. Causal Relationship Between Immunophenotype and Osteomyelitis

**Figure 2:**
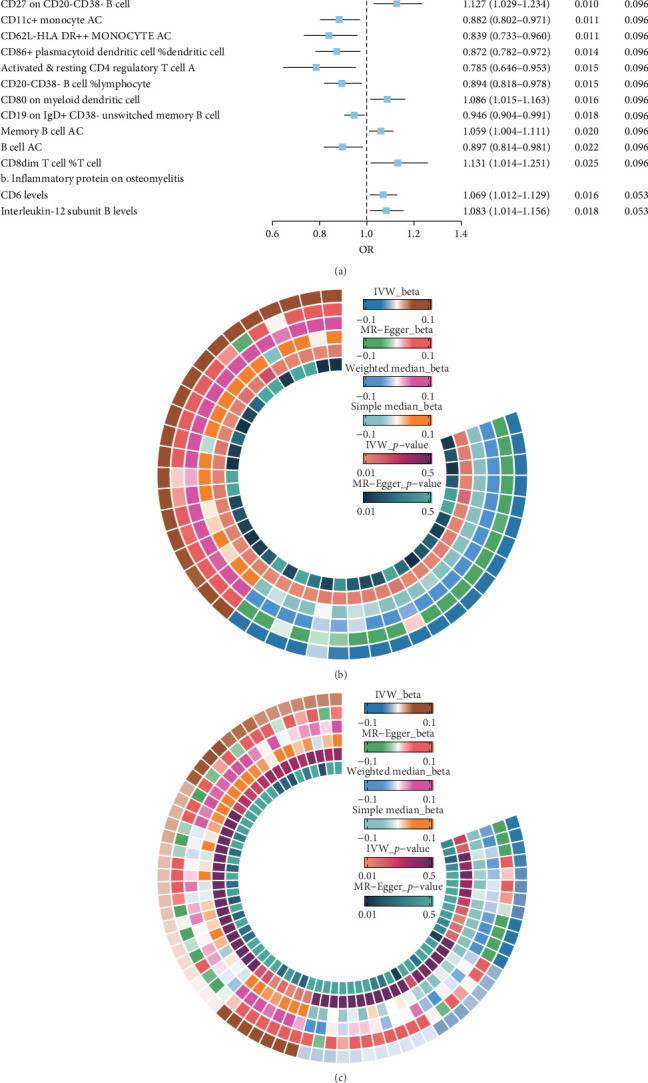
Results of a two-sample MR analysis conducted with genetic data on immune cell phenotypes, plasma inflammatory proteins, and osteomyelitis. (A) Forest plot of MR analysis results; (B) circular heatmap of sensitivity analysis results for immunocyte phenotype-related tests (only immunocyte phenotypes with *p* < 0.05 in the MR analysis are shown); (C) circular heat map of sensitivity analysis results for plasma inflammatory protein-related tests (results for all 70 plasma inflammatory proteins).

**Figure 3:**
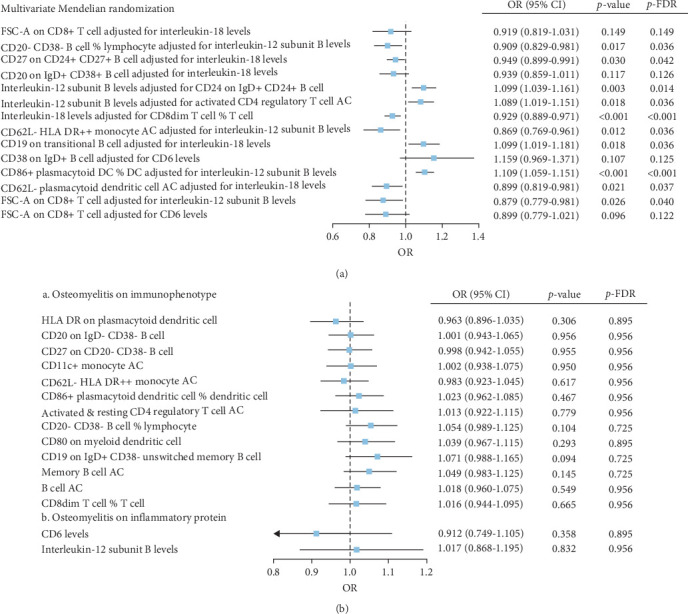
Results of multivariate and reverse MR analysis. (A) Forest plot of multivariate Mendelian randomization analysis carried out on results with *p* < 0.05; (B) reverse Mendelian randomized forest plots for positive outcomes.

The IVW model demonstrated that higher levels of HLA-DR on plasmacytoid dendritic cells (DCs) (OR = 1.147, 95% CI: 1.058–1.243, *p* = 0.001), CD27 on CD20− CD38− B cells (OR = 1.127, 95% CI: 1.058–1.243, *p* = 0.010), CD80 on myeloid DCs (OR = 1.086, 95% CI: 1.015–1.163, *p* = 0.016), memory B cell AC (OR = 1.059, 95% CI: 1.004–1.111, *p* = 0.020), and CD8dim T cell %T cell (OR = 1.131, 95% CI: 1.014–1.251, *p* = 0.025) were associated with an increased risk of developing OM. In contrast, eight immune cell phenotypes, including CD20 on IgD− CD38− B cells, were associated with a reduced risk of OM. However, after FDR correction, only HLA-DR on plasmacytoid DCs and CD20 on IgD− CD38− B cells remained significantly correlated with OM (Figure 2A). Sensitivity analyses using additional MR models confirmed the consistency of effect directions and statistical significance, underscoring the robustness of our findings (Figure 2B). Cochrane's Q test revealed moderate heterogeneity among the IVs for HLA-DR on plasmacytoid DCs (I^2^ = 58.05%, *p* < 0.001) and activated & resting CD4 regulatory T cell AC (I^2^ = 54.95%, *p* = 0.002). No evidence of horizontal pleiotropy was observed in the MR-Egger intercept test (Table 1). However, reverse MR analysis did not identify any causal relationships between OM and the aforementioned immune phenotypes (Figure 3B).

### 3.2. Causal Relationship Between Plasma Inflammation-Associated Proteins and Osteomyelitis

The IVW model also revealed that elevated levels of CD6 (OR = 1.069, 95% CI: 1.012–1.129, *p* = 0.016) and IL-12 subunit B (OR = 1.083, 95% CI: 1.014–1.156, *p* = 0.018) were moderately associated with an increased risk of OM (Figure 2A). Sensitivity analyses using alternative MR models confirmed the direction of effects and significance, further supporting the robustness of the findings (Figure 2C). However, the FDR-corrected negative results indicated that the associations between these two inflammatory proteins and OM only reached marginal significance. Cochrane's Q test and the MR-Egger intercept test did not indicate any significant heterogeneity or horizontal pleiotropy (Table 1). In reverse MR analysis, no causal relationships were observed between OM and levels of either CD6 (OR = 0.912, 95% CI: 0.749–1.105, *p* = 0.358) or IL-12 (OR = 1.017, 95% CI: 0.869–1.195, *p* = 0.832) (Figure 3B).

### 3.3. Interactive Role of ImmunityInflammation in the Pathogenesis of Osteomyelitis

We investigated potential mediating factors by treating immunophenotypes and plasma inflammation-related proteins as both exposures and outcomes. Through mediation MR analysis, five complementary mediation effects and nine competitive mediation effects were identified (Table 2). Among mediators with complementary effects greater than 15%, elevated IL-18 levels mediated the protective effects of increased FSC-A on CD8+ T cells (Proportion = 28.02%, 95% CI: 25.04–30.98) and CD27 on CD24+ CD27+ B cells (Proportion = 15.03%, 95% CI: 12.71–17.45) against OM. Furthermore, reduced levels of IL-12 subunit B mediated the protective effect of CD20− CD38− B cell %lymphocyte against OM (Proportion = 19.21%, 95% CI: 16.58–21.79). Among mediators with competitive effects greater than 15%, increased levels of IL-12 subunit B (Proportion = −16.22%, 95% CI: −13.74–−18.62) and CD6 (Proportion = −17.87%, 95% CI: −15.36–−20.44) competitively inhibited the protective effect of FSC-A on CD8+ T cells against OM. These exposure–outcome combinations with mediating effects warrant further evaluation through MVMR.

In the MVMR analysis, for exposures with complementary mediation effects, CD20− CD38− B cell %lymphocyte remained significant after adjusting for IL-12 subunit B levels (*p* = 0.017). However, the potential significance (at *α* = 0.05) for FSC-A on CD8+ T cells and CD20 on IgD+ CD38+ B cells was lost after adjusting for IL-18. For exposures with competitive mediation effects, increased IL-18 levels (OR = 0.929, 95% CI: 0.889–0.971, *p* < 0.001) after adjusting for CD8dim T cell %T cell, increased CD62L− HLA DR++ monocyte AC levels (OR = 0.869, 95% CI: 0.769–0.961, *p* = 0.012) after adjusting for IL-12 subunit B levels, and increased CD62L− plasmacytoid dendritic cell AC levels (OR = 0.899, 95% CI: 0.819–0.981, *p* = 0.021) were significantly associated with a reduced risk of OM. Conversely, elevated CD19 on transitional B cells (OR = 1.099, 95% CI: 1.019–1.181, *p* = 0.018) after adjusting for IL-18 levels, and increased CD86+ plasmacytoid dendritic cell %Dendritic cell (OR = 1.109, 95% CI: 1.059–1.151, *p* < 0.001) after adjusting for IL-12 subunit B levels, were significantly associated with an increased risk of OM (Figure 3A). This significant association remained after adjusting the *p* values using FDR.

### 3.4. Pathway Enrichment and Protein Interaction Networks of Related Genes

**Figure 4:**
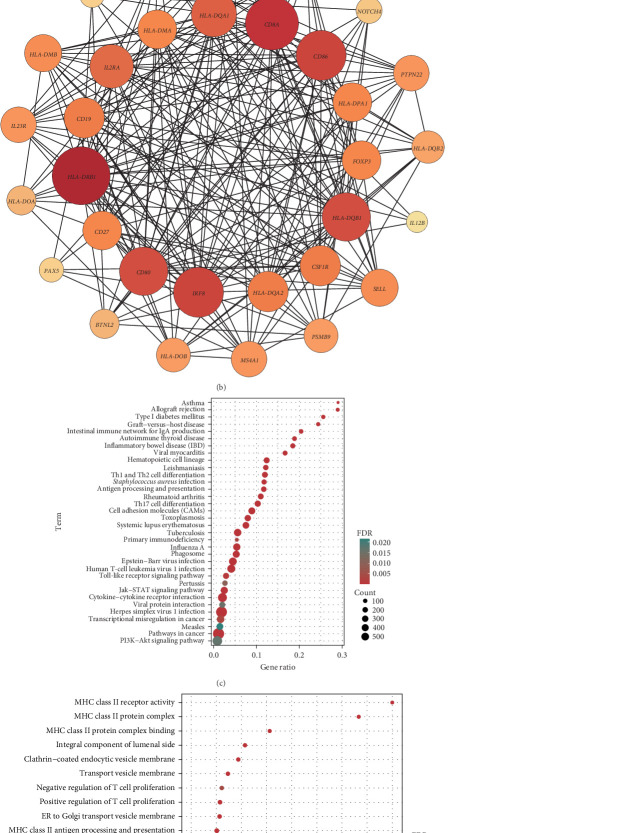
Results of bioinformatics analysis carried out using the identified target genes. (A) Intersecting Venn plots of target genes in different phenotypes; (B) protein–protein interaction network plot of target gene expression products; (C) KEGG pathway enrichment plot of target genes (false discover rate <0.05); (D) GO pathway enrichment plot of target genes (false discover rate <5e–8).

At the genome-wide significance threshold (*α* = 5e−8), a total of 21 OM-associated target genes were identified. Additionally, 18 and 6 target genes were identified for immune cell phenotypes and plasma inflammation-related proteins, respectively. Notably, the IL2RA gene was associated with OM, immune cell phenotypes, and inflammation-related proteins. Furthermore, the HLA-DQA1 and HLA-DRB1 genes were common to both OM and immune cell phenotypes (Figure 4A). The PPI co-occurrence network analysis revealed that proteins expressed by the HLA-DRB1 and CD8A genes exhibited the most significant co-occurrence. The protein expressed by the IL2RA gene, which is associated with all three phenotypes, interacted closely with proteins expressed by key genes such as CD86 and HLA-DRB1, underscoring the complex interaction network between inflammation and immune-related proteins (Figure 4B).

The KEGG pathway enrichment analysis indicated that the target genes were significantly enriched in pathways related to diseases such as asthma, type 1 diabetes, inflammatory bowel disease, and viral myocarditis. In terms of immune regulation, the target genes were significantly enriched in pathways involved in the differentiation of helper T cells, including Th1, Th2, and Th17. Pathways directly related to OM, such as *Staphylococcus aureus* infection, also showed significant enrichment. Moreover, key signaling pathways, including the Jak-STAT, PI3K-Akt, and toll-like receptor signaling pathways, were substantially enriched (Figure 4C). GO enrichment analysis demonstrated significant enrichment in immune-related pathways, particularly involving MHC class II-associated proteins and receptors. T cell-associated receptors and regulatory pathways also showed notable enrichment (Figure 4D).

## 4. Discussion

The objective of this work is to elucidate the role of the immunity–inflammation axis in the pathogenesis of OM and to determine the interplay between immune responses and inflammation. We identified 13 immunophenotypes associated with OM and two related plasma inflammation-associated proteins. By employing a combination of MR and bioinformatics approaches, along with sensitivity analyses such as MR-Egger intercept tests and Cochrane's Q tests, we aimed to more accurately identify pleiotropy and exclude potential measured or unmeasured confounders. This study provides important genetic insights for risk assessment, prevention, and treatment of OM.

Concerning plasma inflammation-associated proteins, we identified elevated levels of CD6 and IL-12 subunit B as significant risk factors for OM. CD6 is a costimulatory molecule located on the surface of T cells, playing a crucial role in T cell activation and immune responses. Elevated CD6 levels can enhance the activation and proliferation of T cells, particularly Th17 cells [27, 28]. Th17 cells produce pro-inflammatory cytokines, such as IL-17, which can lead to increased inflammation and tissue damage [29], hallmarks of OM. Furthermore, the interaction between CD6 and its ligand ALCAM is vital for the recruitment of T cells to inflamed tissues, exacerbating the inflammatory response [30]. IL-12 is a heterodimeric cytokine composed of p35 and p40 subunits, with the p40 subunit encoded by the IL-12B gene. Primarily produced by antigen-presenting cells, IL-12 is critical for the differentiation of naïve T cells into Th1 cells [31]. Th1 cells secrete gamma interferon (IFN-*γ*), which activates macrophages and enhances their ability to eliminate intracellular pathogens [32]. However, excessive IL-12 can result in an overactive immune response, leading to increased production of pro-inflammatory cytokines and further tissue damage [31]. This exacerbated inflammatory state contributes to the onset and progression of OM. Moreover, the concurrent elevation of CD6 and IL-12B levels creates a highly inflammatory environment; this synergistic effect may lead to sustained and excessive inflammation, resulting in severe tissue damage and the development of OM.

Of particular interest, studies related to immunophenotypes indicated that elevated levels of HLA-DR on plasmacytoid dendritic cells (pDCs) emerged as a risk factor for OM. pDCs are crucial antigen-presenting cells within the immune system, primarily responsible for recognizing pathogens and initiating immune responses [33]. HLA-DR is a major class II molecule of the major histocompatibility complex (MHC) found on the surface of pDCs, which participates in the presentation of antigens to T cells [34]. When HLA-DR levels are elevated, the antigen-presenting capacity of pDCs is enhanced, leading to stronger T cell activation and proliferation. This amplified immune response can result in excessive inflammation and tissue damage, particularly pronounced in OM. Additionally, an increase in the percentage of CD8dim T cells within the T cell population represents a significant risk factor for OM. CD8dim T cells, characterized by lower levels of CD8 expression, may negatively influence immune regulation, resulting in an exaggerated immune response to infections. Simultaneously, the cytotoxic function of CD8dim T cells may be compromised, rendering them less effective in clearing infectious pathogens [35]. This could lead to the persistence of pathogens within bone tissue, further triggering and exacerbating OM. The proportion of CD8dim T cells, in conjunction with other characteristic changes in immune cells, may serve as a valuable tool for the early screening and diagnosis of OM. Notably, a study by Ye et al. [36] published in 2024 employed a similar MR approach to explore the associations between pDCs and other diseases, suggesting that the genetic correlations of pDCs with multiple diseases warrant broader attention.

Interestingly, our findings indicate that an increase in B cell activation contributes to a reduced risk of OM, while an increase in memory B cell activation is associated with a higher incidence of the disease. This discrepancy may stem from their distinct roles within the immune system, which dictate their differing impacts on OM risk. Increased B cell activation primarily reduces infection risk by enhancing early immune responses, whereas the rise in memory B cell activation may elevate the risk of OM through excessive immune reactions and chronic inflammation [37, 38]. A study by Surendar et al. [39], focusing on the immune characteristics of patients with osteomyelitis, also reported a significant increase in members of the follicular helper T cell (Tfh) family, as well as in classical and typical memory B cells among individuals with OM. Moreover, a strong correlation between memory B cells and Tfh cells was observed, supporting the reliability of our findings. In our KEGG enrichment analysis, we similarly observed significant functional enrichment of related target genes in Th cell differentiation. Th2 cells promote antibody production by B cells through the secretion of cytokines such as IL-4 and IL-6 [40], which explains why previous researchers have proposed diagnostic strategies for OM that incorporate immune cells and cytokines [11]. The imbalance in Th cell differentiation and function plays a crucial role in the onset and progression of osteomyelitis, making an understanding of these mechanisms essential for developing new therapeutic strategies.

In our mediation analysis, the protective effect of IL-18-mediated FSC-A on CD8+ T cells against OM exhibited the highest mediation proportion. Elevated levels of IL-18 are associated with a reduced risk of OM, and there is a positive correlation between the levels of FSC-A on CD8+ T cells and IL-18. IL-18, a pro-inflammatory cytokine belonging to the IL-1 family, plays a significant role in enhancing both innate and adaptive immune responses. By binding to its receptors (IL-18R*α* and IL-18R*β*), IL-18 activates downstream signaling pathways that promote the production of IFN-*γ*, which is crucial for enhancing the survival and functionality of CD8+ T cells [41]. Forward scatter area (FSC-A) is a parameter used in flow cytometry to measure cell size; an increased FSC-A value in CD8+ T cells typically indicates that these cells are in an activated state, possessing greater proliferative capacity and functionality [42]. Based on our findings, we hypothesize that IL-18 strengthens the immune defense system by enhancing the survival, proliferation, and functionality of CD8+ T cells. This bolstered immune response can more effectively eliminate infectious pathogens, thereby reducing chronic inflammation and tissue damage, ultimately lowering the risk of osteomyelitis.

Long et al. [43] conducted a MR study utilizing immune cell phenotypes and osteomyelitis, employing a more stringent threshold of *α* = 5e–6 to filter instrumental variables (IVs), which resulted in the exclusion of many immune-related SNPs. However, this study did not adequately detail the role of the immunity–inflammation axis in the pathogenesis of osteomyelitis. Additionally, the absence of rigorous multiple testing corrections and well-designed sensitivity analyses increases the likelihood of false positives. The study used GWAS data on osteomyelitis from FinnGen R5, which included only 842 patients, highlighting the need for further research to update this dataset. Similar to our study, Long et al. [43] observed that increases in memory B cells and HLA-DR on plasmacytoid dendritic cells may enhance susceptibility to OM, while decreased frequencies of CD62L monocytes and CD86 plasmacytoid dendritic cells were linked to a reduced risk of OM. Even after limiting our sample population, the characteristic alterations in immune cells continued to affect the risk of OM, further validating the robustness of our findings. Compared with this conventional MR study, our research uniquely highlights the role of the immunity–inflammation axis in the pathogenesis of OM, integrating multivariable MR analysis, mediation analysis, and various bioinformatics approaches to provide a comprehensive explanation of immune–inflammation network interactions. This work offers new insights for the prevention, diagnosis, and targeted treatment of osteomyelitis.

However, it is essential to acknowledge the inherent limitations of this study. Similar to previous MR studies focused on immune cells, the current large-scale GWAS investigating immune cells and plasma inflammatory proteins has primarily been conducted in European populations. Consequently, our findings may have racial limitations; given the genetic heterogeneity among different ethnic groups, the relationship between the immune–inflammation axis and OM may differ in non-European populations. Additionally, it is important to note that MR and bioinformatics analyses are observational and causal inference approaches based solely on genetic data, which cannot substitute for clinical trials in the objective domain. Finally, due to methodological constraints, while we considered the life stages of populations in selecting IVs, the temporal aspect of OM development was overlooked, which may not fully account for potential confounding factors. Therefore, we encourage global researchers to refer to the conclusions of our preliminary study and conduct multicenter clinical cohort studies to further investigate the effects of the immune–inflammation axis on OM.

Our study carries significant clinical implications. First, it provides new biomarker references for the early diagnosis and prevention of OM, including CD6, IL12B, HLA-DR on plasmacytoid dendritic cells, and characteristic changes associated with B cells. Moreover, the findings may guide future therapeutic strategies; interventions targeting specific immune cell phenotypes or plasma inflammatory proteins could potentially reduce the risk of OM. Future researchers should undertake multicenter clinical cohort studies to validate these findings and explore their effects in different ethnic groups and populations. Given the complexity of OM, future research may require interdisciplinary collaboration, integrating knowledge from immunology, genetics, bioinformatics, and other fields to comprehensively understand the disease and develop novel therapeutic approaches.

## 5. Conclusion

In conclusion, this study employed a comprehensive approach, combining MR analysis and bioinformatics methods to explore the genetic associations between immune cell phenotypes, plasma inflammatory proteins, and osteomyelitis, as well as their potential roles in the pathogenesis of OM. The results revealed that 13 immune cell phenotypes and 2 plasma inflammatory proteins are associated with altered OM risk. Future research should validate these results in diverse populations and explore therapeutic strategies targeting these biomarkers. Additionally, interdisciplinary collaboration will be crucial for advancing OM treatment. This study lays a foundation for understanding OM pathogenesis and improving patient outcomes.

## Figures and Tables

**Table 1:** Heterogeneity and horizontal pleiotropy of positive results of MR analysis with immunophenotype and inflammatory protein as exposure and Osteomyelitis as outcome.

Trails	Heterogeneity		Pleiotropy		
	I^2^	*p*-Value	EI	SE	*p*-Value
a. Immunophenotype on osteomyelitis					
HLA DR on plasmacytoid dendritic cell	58.05%	<0.001	−0.045	0.023	0.068
CD20 on IgD- CD38- B cell	9.37%	0.325	−0.014	0.020	0.482
CD27 on CD20- CD38- B cell	0%	0.742	−0.017	0.014	0.246
CD11c+ monocyte AC	5.52%	0.391	−0.028	0.028	0.335
CD62L- HLA DR++ monocyte AC	0%	0.791	−0.030	0.025	0.249
CD86+ plasmacytoid dendritic cell %Dendritic cell	0%	0.552	0.026	0.018	0.160
Activated & resting CD4 regulatory T cell AC	54.95%	0.002	−0.034	0.036	0.363
CD20- CD38- B cell %lymphocyte	24.34%	0.143	0.029	0.018	0.118
CD80 on myeloid dendritic cell	0%	0.528	0.006	0.014	0.705
CD19 on IgD+ CD38- unswitched memory B cell	0%	0.607	−0.020	0.023	0.398
Memory B cell AC	0%	0.717	−0.002	0.015	0.916
B cell AC	0%	0.886	0.016	0.015	0.306
CD8dim T cell %T cell	0%	0.960	0.021	0.029	0.469
b. Inflammatory protein on osteomyelitis					
CD6 levels	0%	0.683	0.004	0.025	0.877
Interleukin-12 subunit B levels	0%	0.908	−0.010	0.030	0.742

Abbreviations: AC, absolute cell counts; EI, egger intercept; SE, standard error.

**Table 2:** Interactive mediating effects of immune cell phenotypes and plasma metabolites in the pathogenesis of osteomyelitis.

Exposure	Mediating	Proportion (%)	95% CI	*p*Value	Type
FSC-A on CD8+ T cell	Interleukin-18 levels	28.02	25.04–30.98	<0.001	Complementarity
CD20- CD38- B cell %lymphocyte	Interleukin-12 subunit B levels	19.21	16.58–21.79	<0.001	Complementarity
CD27 on CD24+ CD27+ B cell	Interleukin-18 levels	15.03	12.71–17.45	<0.001	Complementarity
CD20 on IgD+ CD38+ B cell	Interleukin-18 levels	14.12	11.75–16.36	<0.001	Complementarity
Interleukin-12 subunit B levels	CD24 on IgD+ CD24+ B cell	1.44	0.62–2.18	<0.001	Complementarity
Interleukin-12 subunit B levels	Activated & resting CD4 regulatory T cell AC	−6.88	−5.23–−8.59	<0.001	Competitive
Interleukin-18 levels	CD8dim T cell %T cell	−9.20	−7.34–−11.18	<0.001	Competitive
CD62L- HLA DR++ monocyte AC	Interleukin-12 subunit B levels	−9.47	−7.56–−11.44	<0.001	Competitive
CD19 on transitional B cell	Interleukin-18 levels	−10.12	−8.14–−12.14	<0.001	Competitive
CD38 on IgD+ B cell	CD6 levels	−10.67	−8.64–−12.73	<0.001	Competitive
CD86+ plasmacytoid dendritic cell %dendritic cell	Interleukin-12 subunit B levels	−11.95	−9.78–−14.07	<0.001	Competitive
CD62L- plasmacytoid dendritic cell AC	Interleukin-18 levels	−12.48	−10.3–−14.68	<0.001	Competitive
FSC-A on CD8+ T cell	Interleukin-12 subunit B levels	−16.22	−13.74–−18.62	<0.001	Competitive
FSC-A on CD8+ T cell	CD6 levels	−17.87	−15.36–−20.44	<0.001	Competitive

Abbreviations: AC, absolute cell counts; CI, confidence interval.

## Data Availability

The data that support the findings of this study are openly available. GWAS data for osteomyelitis were obtainedfrom FinnGen consortium (https://r10.finngen.fi/) and IEU Open GWAS Project (https://gwas.mrcieu.ac.uk/).

## Supporting Information

Supporting Information Table S1. STROBE-MR checklist of recommended items to address in reports of Mendelian randomization studies.
