# Supplementary material for: Influence of Altered Immune-Inflammatory Axis on the Risk of Osteomyelitis and Its Network Interaction Effect in European Population
Source: Mediators Inflamm. 2025 Apr 23;2025:5707884. doi: 10.1155/mi/5707884 (PMC12043436; doi:10.1155/mi/5707884)
Supplement: Supporting Information — Table S1. STROBE-MR checklist of recommended items to address in reports of Mendelian randomization studies. [file 5707884.f1.docx]

**Table S1.** STROBE-MR checklist of recommended items to address in reports of Mendelian randomization studies. ^1^ ^2^

| **Item No.** | **Section** | **Checklist item** | **Page No.** | **Relevant text from manuscript** |
| --- | --- | --- | --- | --- |
| 1 | **TITLE and ABSTRACT** | Indicate Mendelian randomization (MR) as the study’s design in the title and/or the abstract if that is a main purpose of the study | 1 | To explore … using Mendelian randomization (MR) and bioinformatics. |
|  | **INTRODUCTION** |  |  |  |
| 2 | **Background** | Explain the scientific background and rationale for the reported study. What is the exposure? Is a potential causal relationship between exposure and outcome plausible? Justify why MR is a helpful method to address the study question | 3 | Osteomyelitis (OM) is a severe inflammatory …a deeper understanding of OM pathogenesis is crucial for the development of targeted prevention and treatment strategies |
| 3 | **Objectives** | State specific objectives clearly, including pre-specified causal hypotheses (if any). State that MR is a method that, under specific assumptions, intends to estimate causal effects | 4 | In this study, we employed MR …relationships between 731 immune cell phenotypes and 70 plasma inflammation-related proteins and OM. |
|  | **METHODS** |  |  |  |
| 4 | **Study design and data sources** | Present key elements of the study design early in the article. Consider including a table listing sources of data for all phases of the study. For each data source contributing to the analysis, describe the following: |  |  |
|  | a) | Setting: Describe the study design and the underlying population, if possible. Describe the setting, locations, and relevant dates, including periods of recruitment, exposure, follow-up, and data collection, when available. | 5 | The immune cell data were sourced from a 2020 GWAS conducted by Orrù et al…. |
|  | b) | Participants: Give the eligibility criteria, and the sources and methods of selection of participants. Report the sample size, and whether any power or sample size calculations were carried out prior to the main analysis | 6 | The genetic data for OM were sourced from the FinnGen consortium … comprising 1,881 OM patients and 391,037 healthy controls… |
|  | c) | Describe measurement, quality control and selection of genetic variants | 7 | To ensure the robustness and reliability of the MR analysis, we applied the following selection criteria for IVs… |
|  | d) | For each exposure, outcome, and other relevant variables, describe methods of assessment and diagnostic criteria for diseases | 6 | Cases were identified using the International Classification of Diseases, 10th Revision codes. |
|  | e) | Provide details of ethics committee approval and participant informed consent, if relevant | 5 | No additional ethical approval was required. |
| 5 | **Assumptions** | Explicitly state the three core IV assumptions for the main analysis (relevance, independence and exclusion restriction) as well assumptions for any additional or sensitivity analysis | 7 | First, to associate SNPs with the exposure, we set the significance threshold for IVs at *p* < 1×10⁻⁵. Additionally… |
| 6 | **Statistical methods: main analysis** | Describe statistical methods and statistics used |  |  |
|  | a) | Describe how quantitative variables were handled in the analyses (i.e., scale, units, model) | 7 | See “Statistical Models and Approaches” Section |
|  | b) | Describe how genetic variants were handled in the analyses and, if applicable, how their weights were selected | 7 | See “Statistical Models and Approaches” Section |
|  | c) | Describe the MR estimator (e.g. two-stage least squares, Wald ratio) and related statistics. Detail the included covariates and, in case of two-sample MR, whether the same covariate set was used for adjustment in the two samples | 7 | We used the inverse variance weighted (IVW) method to evaluate the association between exposures and outcomes… |
|  | d) | Explain how missing data were addressed | 7 | See “Statistical Models and Approaches” Section |
|  | e) | If applicable, indicate how multiple testing was addressed | 8 | See “Mediation and Multivariable Analyses” Section |
| 7 | **Assessment of assumptions** | Describe any methods or prior knowledge used to assess the assumptions or justify their validity | 7 | See “Statistical Models and Approaches” Section |
| 8 | **Sensitivity analyses and additional analyses** | Describe any sensitivity analyses or additional analyses performed (e.g. comparison of effect estimates from different approaches, independent replication, bias analytic techniques, validation of instruments, simulations) | 7 | Heterogeneity was assessed using Cochrane’s Q test and quantified by the I² statistic… |
| 9 | **Software and pre-registration** |  |  |  |
|  | a) | Name statistical software and package(s), including version and settings used | 8 | All statistical analyses were conducted using R version 4.3.2 and the “TwoSampleMR” package |
|  | b) | State whether the study protocol and details were pre-registered (as well as when and where) | -- | Not applicable. |
|  | **RESULTS** |  |  |  |
| 10 | **Descriptive data** |  |  |  |
|  | a) | Report the numbers of individuals at each stage of included studies and reasons for exclusion. Consider use of a flow diagram | -- | Not applicable. |
|  | b) | Report summary statistics for phenotypic exposure(s), outcome(s), and other relevant variables (e.g. means, SDs, proportions) | 9-10 | The IVW model demonstrated that higher levels of HLA-DR on… |
|  | c) | If the data sources include meta-analyses of previous studies, provide the assessments of heterogeneity across these studies | -- | Not applicable. |
|  | d) | For two-sample MR:  i.  Provide justification of the similarity of the genetic variant-exposure associations between the exposure and outcome samples  ii.  Provide information on the number of individuals who overlap between the exposure and outcome studies | i.9-10  ii.6-7 | The GWAS datasets used in this study were of European ancestry and free from population overlap, allowing for the appropriate implementation of MR analyses… |
| 11 | **Main results** |  |  |  |
|  | a) | Report the associations between genetic variant and exposure, and between genetic variant and outcome, preferably on an interpretable scale | 9-11 | See “Causal relationship between immunophenotype and osteomyelitis” Section etc. |
|  | b) | Report MR estimates of the relationship between exposure and outcome, and the measures of uncertainty from the MR analysis, on an interpretable scale, such as odds ratio or relative risk per SD difference | 9-11 | See “Causal relationship between immunophenotype and osteomyelitis” Section etc. |
|  | c) | If relevant, consider translating estimates of relative risk into absolute risk for a meaningful time period | 9-11 | See “Causal relationship between immunophenotype and osteomyelitis” Section etc. |
|  | d) | Consider plots to visualize results (e.g. forest plot, scatterplot of associations between genetic variants and outcome versus between genetic variants and exposure) | 9-11 | See Figure 2-3 |
| 12 | **Assessment of assumptions** |  |  |  |
|  | a) | Report the assessment of the validity of the assumptions | 9-11 | See “Causal relationship between immunophenotype and osteomyelitis” Section etc. |
|  | b) | Report any additional statistics (e.g., assessments of heterogeneity across genetic variants, such as *I^2^*, Q statistic or E-value) | 9-11 | Cochrane’s Q test revealed moderate heterogeneity among the IVs for… |
| 13 | **Sensitivity analyses and additional analyses** |  |  |  |
|  | a) | Report any sensitivity analyses to assess the robustness of the main results to violations of the assumptions | 9-11 | See “Causal relationship between immunophenotype and osteomyelitis” Section etc. |
|  | b) | Report results from other sensitivity analyses or additional analyses | 9-11 | See “Causal relationship between immunophenotype and osteomyelitis” Section etc. |
|  | c) | Report any assessment of direction of causal relationship (e.g., bidirectional MR) | 9-11 | See Figure 3. |
|  | d) | When relevant, report and compare with estimates from non-MR analyses | 17 | Long et al. observed that increases in memory B cells and HLA-DR on plasmacytoid dendritic cells… |
|  | e) | Consider additional plots to visualize results (e.g., leave-one-out analyses) | 9-11 | See Figure 2. |
|  | **DISCUSSION** |  |  |  |
| 14 | **Key results** | Summarize key results with reference to study objectives | 13 | The objective of this work is to elucidate… |
| 15 | **Limitations** | Discuss limitations of the study, taking into account the validity of the IV assumptions, other sources of potential bias, and imprecision. Discuss both direction and magnitude of any potential bias and any efforts to address them | 17-18 | However, it is essential to acknowledge the inherent limitations of this study… |
| 16 | **Interpretation** |  |  |  |
|  | a) | Meaning: Give a cautious overall interpretation of results in the context of their limitations and in comparison with other studies | 16-17 | Long et al. [40] conducted a Mendelian randomization study utilizing immune cell phenotypes and osteomyelitis… |
|  | b) | Mechanism: Discuss underlying biological mechanisms that could drive a potential causal relationship between the investigated exposure and the outcome, and whether the gene-environment equivalence assumption is reasonable. Use causal language carefully, clarifying that IV estimates may provide causal effects only under certain assumptions | 13-14 | CD6 is a costimulatory molecule located on the surface of T cells, playing a crucial role…Moreover, the concurrent elevation of CD6 and IL-12B levels creates a highly inflammatory environment. |
|  | c) | Clinical relevance: Discuss whether the results have clinical or public policy relevance, and to what extent they inform effect sizes of possible interventions | 18 | Our study carries significant clinical implications… |
| 17 | **Generalizability** | Discuss the generalizability of the study results (a) to other populations, (b) across other exposure periods/timings, and (c) across other levels of exposure | 18 | the relationship between the immune-inflammation axis and OM may differ in non-European populations… |
|  | **OTHER INFORMATION** |  |  |  |
| 18 | **Funding** | Describe sources of funding and the role of funders in the present study and, if applicable, sources of funding for the databases and original study or studies on which the present study is based | 20 | This study was supported by… |
| 19 | **Data and data sharing** | Provide the data used to perform all analyses or report where and how the data can be accessed, and reference these sources in the article. Provide the statistical code needed to reproduce the results in the article, or report whether the code is publicly accessible and if so, where | 19 | The data that support the findings of this study are openly available… |
| 20 | **Conflicts of Interest** | All authors should declare all potential conflicts of interest | 19 | All authors declare that they have no conflicts of interest… |

This checklist is copyrighted by the Equator Network under the Creative Commons Attribution 3.0 Unported (CC BY 3.0) license.

1. Skrivankova VW, Richmond RC, Woolf BAR, Yarmolinsky J, Davies NM, Swanson SA, et al. Strengthening the Reporting of Observational Studies in Epidemiology using Mendelian Randomization (STROBE-MR) Statement. JAMA. 2021;under review.

2. Skrivankova VW, Richmond RC, Woolf BAR, Davies NM, Swanson SA, VanderWeele TJ, et al. Strengthening the Reporting of Observational Studies in Epidemiology using Mendelian Randomisation (STROBE-MR): Explanation and Elaboration. BMJ. 2021;375:n2233.
